# Prognostic Significance of NLR, PLR, LMR and Tumor Infiltrating T Lymphocytes in Patients Undergoing Surgical Resection for Hilar Cholangiocarcinoma

**DOI:** 10.3389/fonc.2022.908907

**Published:** 2022-06-03

**Authors:** Zhi-qiang Lin, Chi Ma, Wen-zhuo Cao, Zhen Ning, Guang Tan

**Affiliations:** ^1^Department of General Surgery, The First Affiliated Hospital of Dalian Medical University, Dalian, China; ^2^Health Science Center, East China Normal University, Shanghai, China; ^3^Liaoning Key Laboratory of Molecular Targeted Drugs in Hepatobiliary and Pancreatic Cancer, Dalian Medical University, Dalian, China

**Keywords:** hilar cholangiocarcinoma, NLR, PLR, LMR, tumor-infiltrating lymphocytes

## Abstract

**Objective:**

This study evaluated the prognostic significance of preoperative neutrophil to lymphocyte ratio (NLR), platelet to lymphocyte ratio (PLR), lymphocyte to monocyte ratio (LMR) and tumor-infiltrating lymphocytes (TILs), and whether these preoperative blood inflammatory indicators were associated with TILs in hilar cholangiocarcinoma (HCCA).

**Methods:**

A total of 76 patients with HCCA who underwent radical resection were included. Data on their clinicopathologic characteristics, perioperative features, and survival outcomes were analyzed. The optimal cutoff levels for the NLR, PLR and LMR were defined by using the web application Cut-off Finder. The densities of specific immune cells (CD3^+^, CD4^+^, CD8^+^) within the tumor microenvironment were examined by immunohistochemical. The association of the number of CD3^+^, CD4^+^ and CD8^+^ T cells infiltration in the local tumor microenvironment with preoperative NLR, PLR and LMR level was analyzed. Survival curves were calculated using the Kaplan–Meier estimate. Univariate and multivariate logistic regression models were used to identify factors associated with overall survival.

**Results:**

The optimal cutoff value of preoperative NLR, PLR and LMR was 2.00, 117.60, and 4.02, respectively. NLR was significantly negatively correlated with CD3^+^ and CD8^+^ T cell infiltration, but not with CD4^+^ T cells. PLR had no correlation with CD3^+^, CD4^+^, or CD8^+^ T cell infiltration, while LMR had a significantly positive correlation with CD3^+^ T cells infiltration but not with CD4^+^ or CD8^+^ T cells. In the multivariate logistic regression model, T stage, lymph node metastasis, CA19-9 and LMR were independent risk factors associated with overall survival (OS). Survival curves indicated that HCCA patients with low CD3^+^ T cells infiltration and low preoperative LMR live shorter than others.

**Conclusions:**

LMR played as an independent factor for predicting the survival in patients with HCCA after R0 radical resection. A high LMR was associated with an accumulation of CD3^+^ T cells in HCCA.

## Introduction

Cholangiocarcinoma is a malignant tumor of the mucosal epithelium of the biliary system that accounts for about 3% of all gastrointestinal malignancies (1). It is classified anatomically into three subtypes: intrahepatic cholangiocarcinoma, hilar cholangiocarcinoma, and distal cholangiocarcinoma. The biliary tract tumor with the worst prognosis is hilar cholangiocarcinoma (HCCA), which refers to primary malignant tumors arising in the common hepatic duct, the left and right hepatic ducts, and their confluence. The bile ducts in the hilar region have a unique anatomical position, and the tumor is prone to infiltrating the surrounding blood vessels, nerves, and lymph nodes. Radical resection is the initial and most successful treatment for HCCA. However, the 5-year survival rate following surgery is just approximately 30%, and it is less than 10% for patients with lymph node metastases (2). As a result, it is critical to identify useful prognostic indicators following radical resection.

Inflammation has been recognized as a crucial factor in the development and progression of cancer. In the 19th century, Virchow proposed that the formation and growth of malignant tumors were inextricably tied to organismal inflammation (3). Numerous studies in solid tumors have confirmed that both the systemic inflammatory and local inflammatory responses can indicate prognosis (4). Neutrophils are the most abundant leukocyte component in human blood circulation, which can enter the tumor microenvironment and transform into tumor-associated neutrophils to participate in the tumor process under the action of chemokines secreted by tumors. Platelets are also closely related to tumor growth and metastasis. It has been reported that they directly interact with various components in the tumor microenvironment to promote tumor growth and invasion. On the other hand, platelets promote tumor cell proliferation through their derivatives such as PDGF-C and 5-HT. In recent years, neutrophil to lymphocyte ratio (NLR), platelet to lymphocyte ratio (PLR), and lymphocyte to monocyte ratio (LMR) have been found to be significantly correlated with prognosis in a variety of cancers, especially gastrointestinal tumors (5–7). Additionally, tumor-infiltrating lymphocytes (TILs), particularly cytotoxic CD8^+^ T cells, have also been shown to play a major role in antitumor immunity in the cancer microenvironment. Studies have confirmed that the number of TILs correlates with the prognosis of various tumors such as colorectal cancer, gastric cancer, pancreatic cancer, cholangiocarcinoma and lung cancer (8–12). However, the prognostic significance of NLR, PLR, LMR and TILs in HCCA remains unclear. Furthermore, the correlation between peripheral blood inflammatory indicators and TILs in HCCA remains to be further explored.

In this study, we focused on the prognostic significance of preoperative NLR, PLR, LMR and TILs in HCCA. In addition, we also evaluated the relationship between peripheral blood inflammatory indicators and TILs in HCCA.

## Materials and Methods

### Patient Characteristics

This study included 76 patients with HCCA who underwent R0 radical resection at the First Hospital of Dalian Medical University between January 2010 and January 2018. Patients were staged according to the American Joint Committee on Cancer (AJCC) 8th edition and Bismuth staging system. The exclusion criteria for patients were as follows: (a) preoperative radiotherapy and/or chemotherapy; (b) concurrent serious infections and severe cardiovascular disease; (c) received any drugs that altered peripheral blood cell markers, such as antibiotics, hormones, aspirin, clopidogrel within the previous three months; (d) recurrent HCCA; (e) Lewis-negative patients; (f) patients with blood malignancies and multiple myeloma and other malignant tumors; (g) patients who received preoperative traditional Chinese medicine antitumor treatment (considering that traditional Chinese medicine treatment may affect the immune status of patients). The study was approved by the Ethics Committee of the First Affiliated Hospital of Dalian Medical University.

### Hematology Analysis

Hematology analysis included carcinoembryonic antigen (CEA), carbohydrate antigen 19-9 (CA19-9) and morning fasting blood routine and liver function within one week before surgery. The NLR was calculated by dividing the absolute neutrophil count by the absolute lymphocyte count. The PLR was calculated by dividing the absolute platelet count by the absolute lymphocyte count. The LMR was calculated by dividing the absolute lymphocyte count by the absolute monocyte count.

### Immunohistochemistry

The TILs were examined by immunohistochemical staining. The paraffin-embedded HCCA specimens were obtained from the pathology department of the First Affiliated Hospital of Dalian Medical University. Standard IHC staining procedures were performed according to the instructions of the IHC Kit. CD3 (1:150, abcam, ab16669), CD4 (1:200, CST, 48274) and CD8 (1:30, CST, 85336) were used as the primary antibodies. EDTA or citrate solution were used for antigen retrieval depend on antibody instruction. After sealing the sections using neutral resin, the positively stained cells around the tumor cells were evaluated with a microscope in three randomly selected fields at a magnification of × 400 and the average was calculated.

### Statistical Analysis

Optimal cut-off values for NLR, PLR, and LMR were determined by using the web application Cut-off Finder on OS (13), and the cut-off values were used as a basis for grouping them into higher level groups and lower level groups. The chi-square or Fisher exact tests were used to compare categorical variables, as appropriate. Cox proportional hazard regression analysis was used to find predictive predictors. The Kaplan–Meier method was used to estimate the OS, and the log-rank test was used to compare survival curves. Pearson correlation analysis was used to assess the correlation of NLR, PLR, and LMR with tumor-infiltrating CD3^+^, CD4^+^, and CD8^+^ TILs. Groups were considered significantly different at p<0.05. All tests were performed using SPSS software version 22.0.

## Results

### Baseline Characteristics

Initially, 102 patients with HCCA were identified in our center’s clinical database, but ultimately only 76 patients with detailed data and well-preserved tumor specimens were included in this study. As shown in **Table 1**, the proportions of male and female patients were 53.7% and 46.1%, respectively. The mean age of the patients was 60.4 years. 32 patients (42.1%) were in the T1-T2 stage, and 44 patients (57.9%) were in the T3-T4 stage; 33 patients (43.4%) were Bismuth I-II types, and 43 patients (56.6%) were Bismuth III-IV types. There were 13 (17.1%), 39 (51.3%), and 54 (71.1%) patients with liver parenchymal invasion, portal vein invasion, and nerve invasion, respectively. 65 patients (85.5%) had moderate to low differentiation. 50 patients (64.9%) had tumor size < 3 cm. The vast majority of patients (82.9%) had preoperative total bilirubin above 34.2 μmol/L. Finally, the median OS was 29 months. The 1-year and 3-year OS rates were 76.3% and 28.9%, respectively.

**Table 1:** Baseline characteristics.

Variables	No. of patients (%)
Age(year)	
<60	36 (47.4%)
≥60	40 (52.6%)
Gender	
Male	41 (53.7%)
Female	35 (46.1%)
Tumor stage	
T1-T2	32 (42.1%)
T3-T4	44 (57.9%)
Lymph node metastasis	
No	51 (67.1%)
Yes	25 (32.9%)
Liver invasion	
No	63 (82.9%)
Yes	13 (17.1%)
Portal vein invasion	
No	37 (48.7%)
Yes	39 (51.3%)
Neural invasion	
No	22 (28.9%)
Yes	54 (71.1%)
Bismuth-Coretre type	
I-II	33 (43.4%)
III-IV	43 (56.6%)
Tumor differentiation	
Moderate/poor	65 (85.5%)
well	11 (14.5%)
Tumor size (cm)	
<3	50 (64.9%)
≥3	26 (33.8%)
Total bilirubin (μmol/L)	
<34.2	13 (17.1%)
≥34.2	63 (82.9%)

### Associations Between Clinicopathological Characteristics and Preoperative NLR, PLR and LMR

We used the Cut-off Finder web-based tool to find the optimal cut-off values for NLR, PLR, and LMR in this group of patients. The corresponding optimal cut-off values were: NLR = 2.00, PLR = 117.60, and LMR = 4.02. Patients were divided into high level and low level groups according to the cut-off values.

High NLR was significantly associated with age, more advanced T stage, Bismuth III-IV types and CA19-9 levels (**Table 2**). High PLR was significantly associated with better differentiation and a smaller tumor diameter (**Table 2**). Low LMR was significantly associated with more advanced stages, portal vein invasion, and high CEA levels (**Table 2**).

**Table 2:** Characteristics of the patients grouped immune cells and peripheral blood.

Variables	NLR		*P*	PLR		*P*	LMR		*P*	CD3		*P*	CD4		*P*	CD8		*P*
	<2.00	≥2.00		<117.60	≥117.60		<4.02	≥4.02		<93.5	≥93.5		<56.5	≥56.5		<54.5	≥54.5	
Age (year)																		
<60	20	16	0.001	19	17	0.370	20	16	0.175	16	20	0.358	19	17	0.646	22	14	0.066
≥60	7	33		17	23		16	24		22	18		19	21		16	24	
Gender																		
Male	18	23	0.512	21	20	0.467	18	23	0.512	18	23	0.250	21	20	0.818	23	18	0.198
Female	9	26		15	20		18	17		20	15		17	18		15	20	
Tumor stage																		
T1-T2	16	16	0.025	17	15	0.391	5	27	0.003	9	23	0.001	18	14	0.691	9	23	0.001
T3-T4	11	33		19	25		31	13		29	15		20	24		29	15	
Lymph node metastasis																		
No	18	33	0.952	23	28	0.571	23	28	0.571	19	32	0.002	26	25	0.807	18	33	0.001
Yes	9	16		13	12		13	12		19	6		12	13		20	5	
Bismuth-Coretre type																		
I-II	16	17	0.039	17	16	0.526	16	17	0.864	11	22	0.011	13	20	0.105	12	21	0.025
III-IV	11	32		19	24		20	23		27	16		25	18		26	17	
Tumor differentiation																		
Moderate/poor	23	42	0.949	34	31	0.036	30	35	0.606	33	32	0.744	31	34	0.328	33	32	0.744
well	4	7		2	9		6	5		5	6		7	4		5	6	
Tumor size (cm)																		
<3	19	31	0.532	22	28	0.015	26	24	0.262	26	24	0.472	23	27	0.231	25	25	0.688
≥3	8	18		16	10		10	16		12	14		16	10		13	13	
Liver invasion																		
No	22	41	1.000	29	34	0.607	30	33	0.923	33	30	0.361	29	34	0.128	29	34	0.128
Yes	5	8		7	6		6	7		5	8		9	4		9	4	
Neural invasion																		
No	8	14	0.922	8	14	0.220	13	9	0.191	8	14	0.129	12	10	0.613	7	15	0.052
Yes	19	35		28	26		23	31		30	24		26	28		31	23	
Portal vein invasion																		
No	16	21	0.171	17	20	0.809	13	24	0.037	11	26	0.001	18	19	0.818	12	25	0.002
Yes	11	28		19	20		23	16		27	12		20	19		26	13	
CEA (ng/ml)																		
<5.0	22	43	0.457	30	35	0.486	34	31	0.036	32	33	0.744	32	33	0.744	34	31	0.328
≥5.0	5	6		6	5		2	9		6	5		6	5		4	7	
CA19-9 (U/ml)																		
<37	13	12	0.035	14	11	0.291	16	9	0.052	8	17	0.028	14	11	0.464	7	18	0.007
≥37	14	37		22	29		20	31		30	21		24	27		31	20	
Total bilirubin(μmol/L)																		
<34.2	4	9	0.693	7	6	0.546	8	5	0.261	3	10	0.033	9	4	0.128	7	6	0.761
≥34.2	23	40		29	34		28	35		35	28		29	34		31	32	

CEA, carcinoembryonic antigen; CA, 19-9 cancer antigen 19-9; NLR, neutrophil lymphocyte ratio; PLR, platelet lymphocyte ratio; LMR, lymphocyte monocyte ratio.

### Associations Between Clinicopathological Characteristics and TILs in the Local Tumor Microenvironment

**Figure 1:**
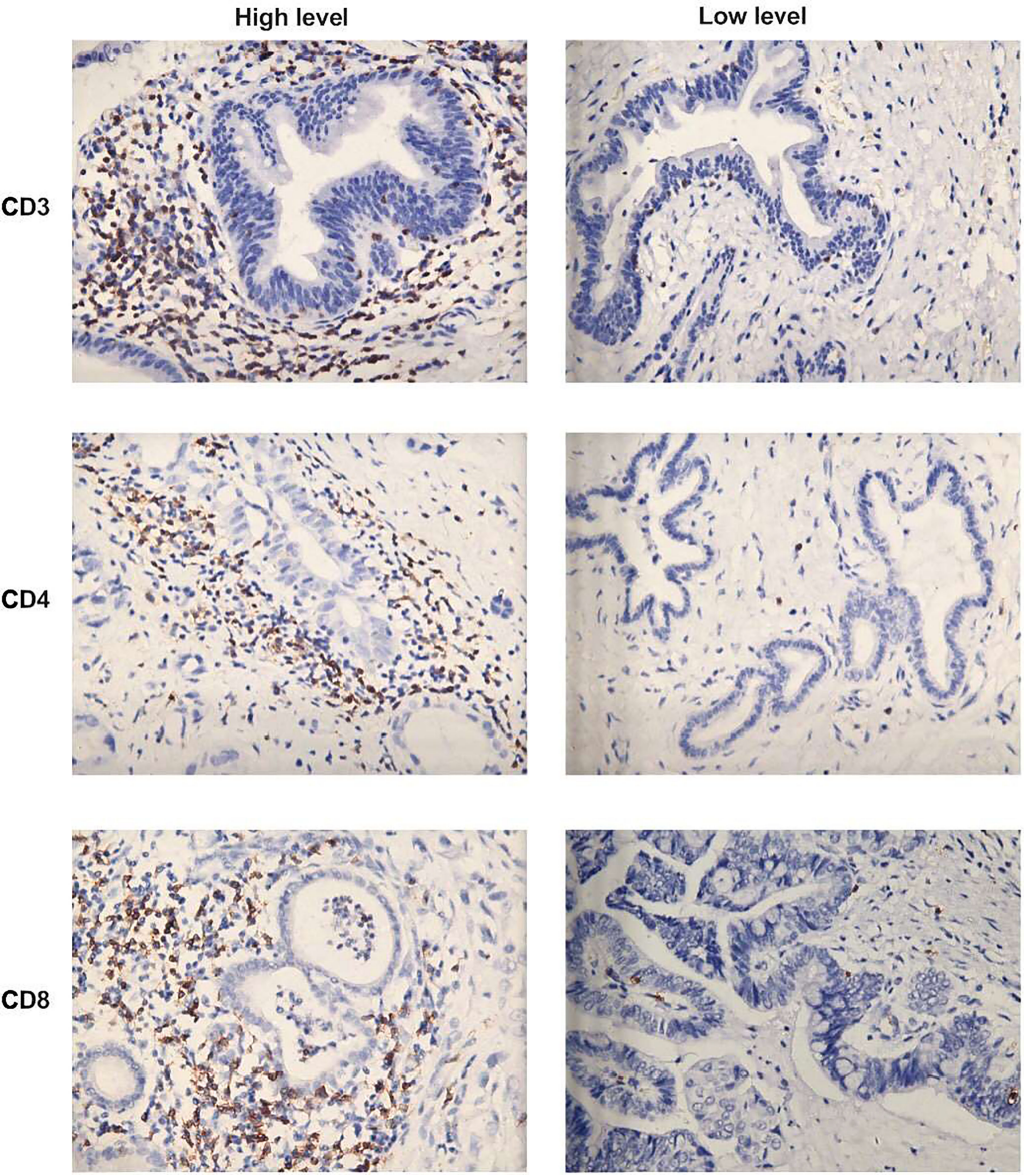
Representative images of immunohistochemical features of high-level and low-level tumor-infiltrating inflammatory cells in hilar cholangiocarcinoma: CD3^+^T cells, CD4^+^ T cells and CD8^+^T cells. (×400 magnification).

The median number of CD3^+^, CD4^+^ and CD8^+^ TILs was 93.5, 56.5 and 54.5, respectively (**Table 2**). **Figure 1** illustrates the representative images of immunohistochemical features of high-level and low-level tumor-infiltrating inflammatory cells in hilar cholangiocarcinoma. All patients were divided into the high group of CD3^+^, CD4^+^ and CD8^+^ TILs and the low group of CD3^+^, CD4^+^ and CD8^+^ TILs based on the median number of TILs, respectively.

Low infiltration of CD3^+^ and CD8^+^ T cells was significantly associated with more advanced T stage, lymph node metastasis, Bismuth III-IV types, portal vein invasion, and high CA19-9 levels (**Table 2**). Furthermore, decreased CD3^+^ T cell infiltration was found to be related to high preoperative total bilirubin levels (**Table 2**). However, infiltration of CD4^+^ T cells was not associated with clinicopathological characteristics (**Table 2**).

### Preoperative NLR, PLR, and LMR Associations With Survival Outcomes

**Figure 2:**
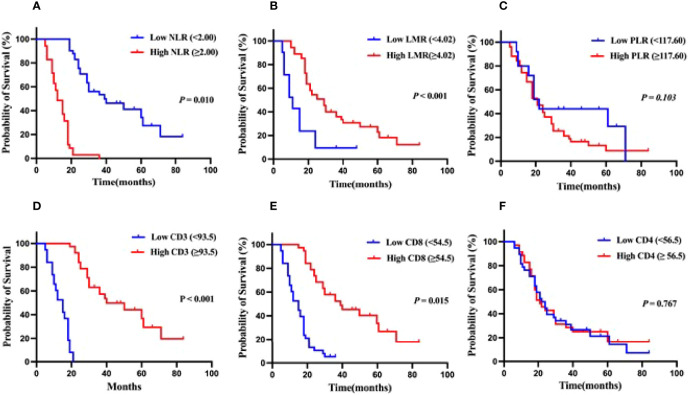
Kaplan-Meier analysis for OS in patients with hilar cholangiocarcinoma.**(A)** OS for patients with different levels of NMR. **(B)** OS for patients with different levels of LMR. **(C)** OS for patients with different levels of PLR. **(D)** OS for patients with different levels of tumor-infiltrating CD3^+^T cells infiltration. **(E)** OS for patients with different levels of tumor-infiltrating CD8^+^T cells infiltration. **(F)** OS for patients with different levels of tumor-infiltrating CD4^+^T cells infiltration.

Patients in the low NLR group had a median OS of 36 months, with 1- and 3-year survival rates of 88.9% and 51.9%, respectively, while patients in the high NLR group had a median OS of 16 months, with 1- and 3-year survival rates of 58.6% and 0.0%, respectively (**Figure 2A**). Additionally, the median OS of patients in the low LMR group was 11 months, with 1 and 3-year survival rates of 52.4% and 9.65%, respectively, and the median OS of patients in the high LMR group was 25 months, with 1 and 3-year survival rates of 89.0% and 36.0%, respectively (**Figure 2B**). However, PLR, on the other hand, did not appear to be related to the patients’ overall survival (**Figure 2C**). According to these findings, HCCA patients with low preoperative NLR and high LMR levels had a better prognosis.

### Association Between TILs in the Local Tumor Microenvironment and Survival Outcomes

Patients with low CD3^+^ T cell infiltration had a median OS of 15 months, with 1- and 3-year survival rates of 52.6% and 0.0%, respectively, and patients with high CD3^+^ T cells infiltration had a median OS of 39 months, with 1- and 3-year survival rates of 89.0% and 57.4%, respectively (**Figure 2D**). Patients with low CD8^+^ T cells infiltration had a median OS of 15 months, with 1- and 3-year survival rates of 51.4% and 5.4%, respectively, whereas those with high CD8^+^ T cells infiltration had a median OS of 37 months, with 1- and 3-year survival rates of 91.0% and 52.4%, respectively (**Figure 2E**). The presence of CD4^+^ T cell infiltration did not appear to be related to the patients’ overall survival (**Figure 2F**). These data indicated that HCCA patients with high levels of local CD3^+^ and CD8^+^ T cell infiltration in the tumor had a better prognosis.

### Correlation Between Preoperative NLR, PLR, LMR and TILs

**Figure 3:**
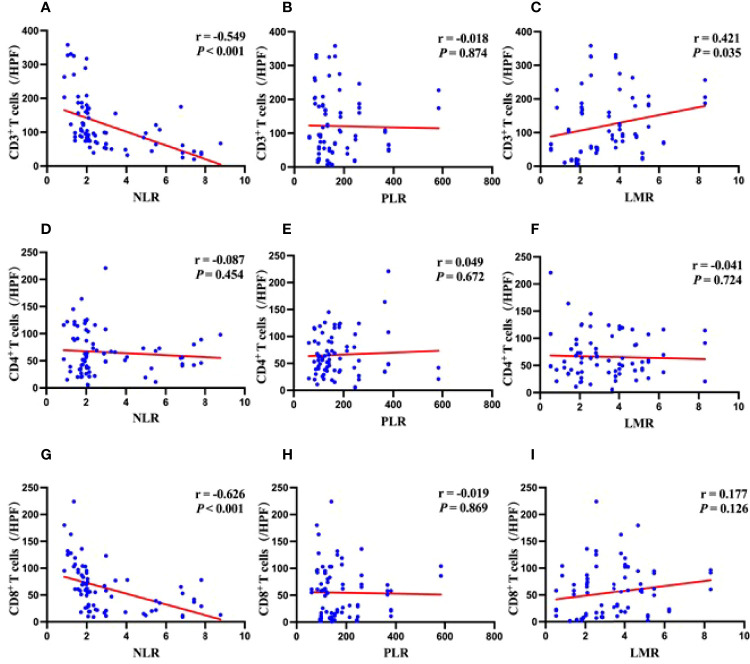
Correlations between systemic inflammation markers and density of immune cells in the tumor microenvironment. **(A)** Correlation between NLR and levels of CD3^+^ T cells infiltration. **(B)** Correlation between PLR and levels of CD3^+^ T cells infiltration. **(C)** Correlation between LMR and levels of CD3^+^ T cells infiltration. **(D)** Correlation between NLR and levels of CD4^+^ T cells infiltration. **(E)** Correlation between PLR and levels of CD4^+^ T cells infiltration. **(F)** Correlation between LMR and levels of CD4^+^ T cells infiltration. **(G)** Correlation between NLR and levels of CD8^+^ T cells infiltration. **(H)** Correlation between PLR and levels of CD8^+^ T cells infiltration. **(I)** Correlation between LMR and levels of CD8^+^ T cells infiltration.

NLR was significantly negatively correlated with CD3^+^ and CD8^+^ T cells infiltration, but not with CD4^+^ T cells (**Figures 3A, D, G**). PLR had no correlation with CD3^+^, CD4^+^, or CD8^+^ T cells infiltration (**Figures 3B, E, H**), while LMR had a significantly positive correlation with CD3^+^ T cells infiltration but not with CD4^+^ or CD8^+^ T cells (**Figures 3C, F, I**).

### Preoperative LMR Is Independent Prognostic Indicator of OS in HCCA Patients

Firstly, univariate analysis identified gender, T-stage, lymph node metastasis, nerve invasion, portal vein invasion, preoperative total bilirubin, Bismuth staging, CA19-9, NLR, LMR, CD3^+^ T cells, and CD8^+^ T cells as factors influencing the prognosis of HCCA patients after radical resection (**Table 3**). Since the outcomes of the appeal indicated that NLR was statistically correlated with CD3^+^ T cells and CD8^+^ T cells, and LMR was significantly correlated with CD3^+^ T cells. Thus CD3^+^ and CD8^+^ T cells were not included in the multifactorial analysis. The rest of the above factors were subsequently included as independent variables in a COX regression proportional risk model for multifactorial analysis. The results revealed that T stage, lymph node metastasis, CA19-9 and LMR were independent predictors of prognosis in HCCA patients after radical resection (**Table 4**). Overall, the above results revealed that more advanced T-stage, portal vein invasion, lymph node metastasis, and low preoperative LMR level are poor prognostic influences for HCCA.

**Table 3:** Univariate analysis for overall survival.

Variables	3-year OS(%)	95% for CI	χ^2^	*P*
Age (year)				
<60	36.8	20.786-36.240	0.081	0.776
≥60	26.3	24.692-38.178		
Gender				
Male	22.0	19.078-31.941	5.712	0.017
Female	37.1	29.449-49.265		
Tumor stage				
T1-T2	56.3	39.720-62.207	29.136	<0.001
T3-T4	13.6	15.708-24.337		
Lymph node metastasis				
No	36.8	30.258-44.609	34.197	<0.001
Yes	12.0	12.201-22.199		
Bismuth-Coretre type				
I-II	48.5	34.778-56.871	33.125	<0.001
III-IV	14.0	17.356-27.388		
Tumor differentiation				
Moderate/poor	27.4	26.219-61.963	2.547	0.110
well	36.4	23.361-34.048		
Tumor size (cm)				
<3	32.1	26.310-43.763	0.501	0.479
≥3	22.2	21.407-35.497		
Liver invation				
No	31.7	26.793-40.869	1.223	0.269
Yes	15.4	17.938-31.447		
Neural invasion				
No	53.8	30.977-54.750	7.075	0.008
Yes	18.5	20.652-32.040		
Portal vein invasion				
No	48.6	36.354-57.150	29.132	<0.001
Yes	10.3	15.349-24.754		
Total bilirubin (μmol/L)				
<34.2	46.2	28.857-56.528	4.334	0.037
≥34.2	25.4	22.806-34.057		
CEA(ng/ml)				
<5.0	29.2	25.165-37.390	0.113	0.737
≥5.0	27.3	17.059-35.487		
CA19-9(U/ml)				
<37	52.0	33.711-57.617	10.674	<0.001
≥37	17.6	19.344-30.094		
NLR				
<2.00	51.9	39.065-55.414	21.352	0.010
≥2.00	0.0	11.451-15.578		
PLR				
<117.60	44.0	27.703-49.363	2.660	0.103
≥117.60	21.2	21.734-34.252		
LMR				
<4.02	9.65	10.025-20.451	30.857	<0.001
≥4.02	36.0	30.261-44.054		
CD3				
<93.5	0.0	11.934-15.172	78.301	<0.001
≥93.5	57.4	41.477-58.111		
CD4				
<56.5	31.1	24.054-40.111	0.087	0.767
≥56.5	26.3	22.931-40.332		
CD8				
<54.5	5.4	25.824-37.727	25.464	0.015
≥54.5	52.4			

CEA, carcinoembryonic antigen; CA, 19-9 cancer antigen 19-9; NLR, neutrophil lymphocyte ratio; PLR, platelet lymphocyte ratio; LMR, lymphocyte monocyte ratio; OS, overall survival; CI, confidence interval.

**Table 4:** Multivariable analysis for overall survival.

Variables	β	SE	Wald	*P*	HR	95.0% for CI
Gender	-0.113	0.366	0.093	0.761	0.893	0.432-1.846
Tumor stage	2.914	0.943	20.521	<0.001	18.428	5.223-65.012
Lymph node metastasis	1.118	0.862	7.099	0.008	2.769	1.309-5.856
Bismuth-Coretre type	0.698	0.677	0.525	0.509	1.384	0.538-2.762
Neural invasion	0.811	0.735	0.725	0.355	2.057	1.098-5.042
Portal vein invasion	0.437	0.440	0.016	0.700	1.070	0.371-3.083
Total bilirubin	0.682	0.661	0.436	0.636	1.219	0.423-5.656
CA19-9	1.184	0.880	7.728	0.005	2.957	1.377-6.350
NLR	0.546	0.535	0.307	0.687	1.147	0.455-4.404
LMR	-1.708	0.889	19.271	<0.001	0.181	0.085-0.389

LMR, lymphocyte monocyte ratio; HR, hazard ratio; CI, confidence interval.

### Preoperative LMR and CD3^+^ T Cells Infiltration in Relation to Survival Outcomes

**Figure 4:**
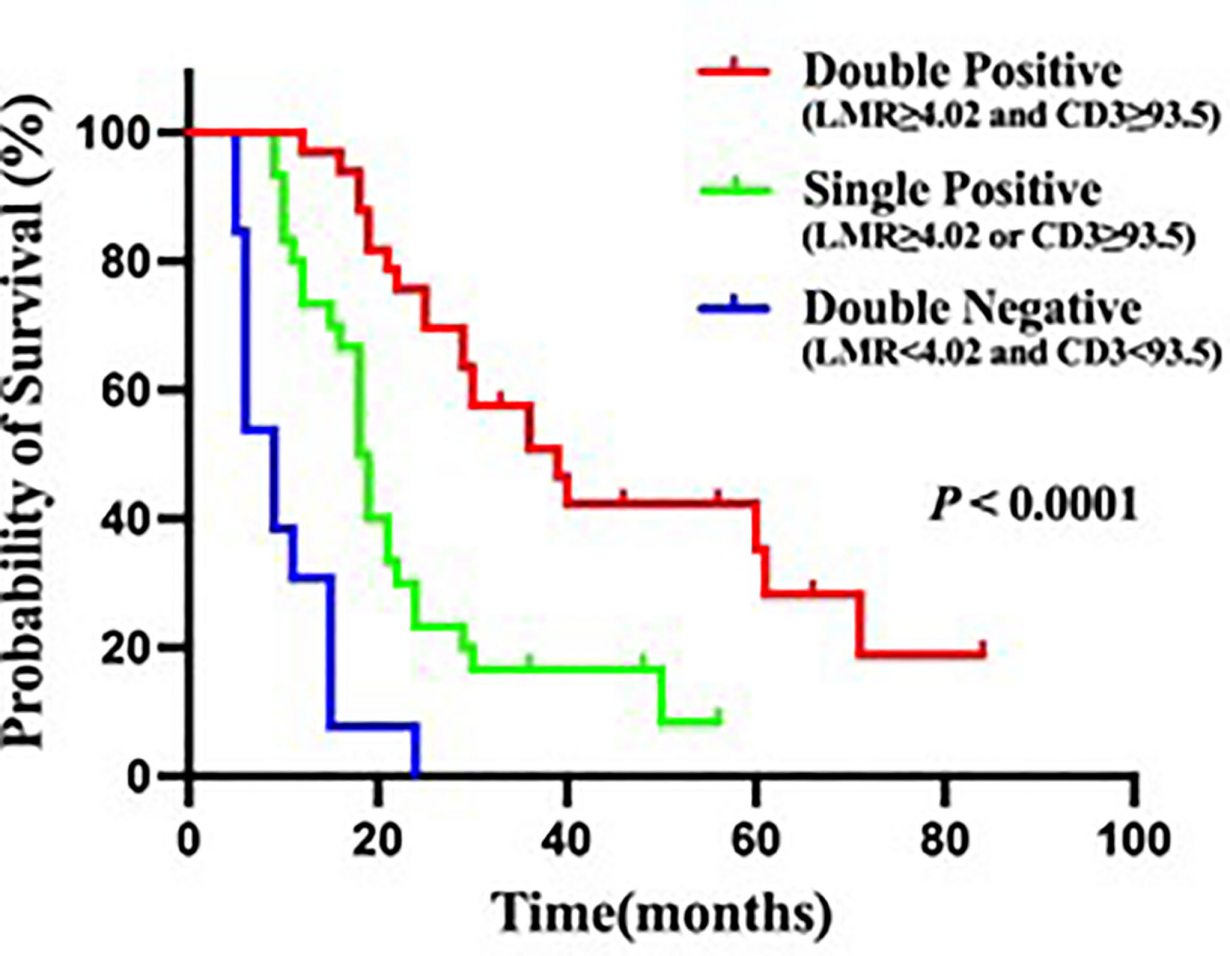
Kaplan-Meier analysis for OS in patients with hilar cholangiocarcinoma. Double positive is LMR≥4.02 and CD3≥93.5, Single positive is LMR≥4.02 or CD3≥93.5, Double negative is LMR<4.02 and CD3<93.5.

Both the CD3^+^ T cells infiltration and LMR could impact prognosis. Therefore, it is important to explore whether combining them can further accurately predict patient survival prognosis. In this study, patients with low CD3^+^ T cells infiltration and low LMR had a median OS of 6 months, with 1- and 3-year survival rates of 33.3% and 0.0%, respectively (**Figure 4**). Whereas patients with high CD3^+^ T cells infiltration or high LMR had a median OS of 18 months, with 1- and 3-year survival rates of 71.0% and 16.1%, respectively (**Figure 4**). Patients with high CD3^+^ T cells infiltration and high LMR had a median OS of 39 months, with 1- and 3-year survival rates of 97% and 45.5% (**Figure 4**). These data suggested that HCCA patients with high levels of local CD3^+^ T cells infiltration in the tumor and high preoperative LMR in the peripheral blood had a better prognosis.

## Discussion

This study showed that preoperative LMR was independent prognostic factor for HCCA after radical resection. In addition, we observed a significant positive correlation between preoperative LMR and tumor-infiltrating CD3+ T cells. Therefore, LMR combined application with the CD3+ T cells infiltration may improve the prognostic accuracy. This is supported by our results, patients with high levels of LMR and CD3+ T cells infiltration had a longer postoperative survival time than others. The combinations of these two biomarkers can be helpful in the clinical practice to address different follow-up schedule and postoperative treatment. High-risk patients after surgery should be closely followed up and treat them more vigorously in order to achieve the best outcomes.

Neutrophils are thought to be a major source of vascular endothelial growth factor (VEGF) (14), which can promote tumor proliferation and neoangiogenesis. In addition, activated neutrophils can suppress anti-tumor immune responses mediated by lymphocytes (15).Under the mediation of tumor chemokines, circulating monocytes are recruited into the tumor microenvironment and differentiate into macrophages, which suppress immune responses and promote tumor metastasis and angiogenesis (16). The most important role of platelets against tumors is to protect tumor cells from the killing effect of NK cells, thus completing the cellular immune escape (17).Lymphocyte count is considered as an indicator of T lymphocyte-mediated antitumor capacity, that is, the host immune response to tumor progression and metastasis is suppressed due to a decrease in lymphocytes (18). Thus, as new systemic inflammatory response markers, NLR, PLR, and LMR are able to show the dynamic balance between inflammation and immunity. High levels of NLR, PLR, and low levels of LMR suggest a strong ability to promote tumor growth and metastasis and a weak anti-tumor immune effect. Previous study suggested that NLR was an independent prognostic factor for advanced hilar cholangiocarcinoma that is operable or receiving adjuvant therapy (19). Lin et al. compared the prognostic value of GPS, mGPS, PI, NLR, PLR, LMR, and PNI in 200 patients with ICC, and although this study showed that all these scores had significant prognostic value, LMR was found to be the best prognostic factor in a multifactorial analysis (20). Similar conclusions were reached by Simone Conci et al. in patients with biliary tract tumors (21).The prognostic value of LMR had also been demonstrated in other malignancies, such as hepatocellular carcinoma, lung cancer, colorectal cancer, etc. (22–24).This study also discovered that patients in the high NLR group or low LMR group had a worse survival prognosis. However, only LMR was identified as independent prognostic markers even in multivariate analysis for OS. A plausible explanation supporting our findings may be the key role played by monocytes in maintaining the chronic inflammatory process and influencing tumor growth.

Tumor cells are often surrounded by infiltrating inflammatory cells, including lymphocytes and neutrophils, etc. It is well recognized that lymphocytes constitute one of the most important effector mechanisms of anti-tumor immunity. T lymphocytes, the hallmark of cell-mediated adaptive immunity are considered essential in tumor immunosurveillance (25). Previous research has shown that the level of T-lymphocyte infiltration in the tumor microenvironment was strongly related to patient prognosis (26, 27). Ryota Tanaka found that the OS and RFS of patients with high tumor-infiltrating CD8^+^ T cells were longer than those with low ones in biliary tract cancers (28).Y Zhang et al. revealed that the density of CD3^+^ T cells infiltration in the tumor microenvironment was positively correlated with OS (29). According to our findings, low-density CD3^+^ T cells and CD8^+^ T cells infiltration were associated with shorter survival time in HCCA. On the one hand, it may be related to a weakened anti-tumor immune capacity. On the other hand, the correlation of low CD3^+^ T cells and CD8^+^ T cells infiltration with indicators of advanced disease may also explain this phenomenon. These results indicated that tumor-infiltrating CD3^+^ T cells and CD8^+^ T cells in HCCA play a key role in tumor immunity in the cancer microenvironment and influence the survival prognosis of patients.

The relationship between the systemic immune response and the tumor microenvironment has not been fully elucidated. Previous studies in colon and gastric cancers have found that NLR and LMR may evaluate lymphocyte infiltration in the tumor microenvironment. For example, a study by Guo et al. found that the higher LMR, the more CD3^+^ T cells infiltration was present in colon cancer tissues (30). Choi et al. also found a negative correlation between NLR and CD4^+^ T cells infiltration in gastric cancer tissues (31). In biliary tract cancers, Ryota Tanaka also found that NLR and CD8^+^ T cells infiltration were negatively correlated. So, NLR could predict CD8^+^ T cells in the tumor microenvironment (28). The present study elucidated that NLR was statistically negatively correlated with tumor-infiltrating CD3^+^ T cells and CD8^+^ T cells, and LMR was significantly positively correlated with tumor-infiltrating CD3^+^ T cells. These results could explain the poor prognosis associated with NLR and LMR in cancer patients. In addition, we also discovered that the density of CD3+ T cells infiltration was decreased in low-LMR patients, who had a worse prognosis. In summary, we proposed that LMR and NLR maybe used to ascertain the immunological status in the tumor microenvironment. Importantly, these markers could be easily calculated in the peripheral blood, avoiding the use of invasive and complex operations to evaluate the tumor immune microenvironment. This information could be highly valuable for selection of patients who will most likely benefit from cancer immunotherapy. However, the underlying reason explaining why blood markers were associated with the density of TILs requires further investigation.

Nevertheless, the present study has a few limitations. Firstly, this was a single-center retrospective study with a small sample size; secondly, only overall survival time was analyzed in this study, not recurrent metastases; thirdly, patients with non-surgical or palliative surgery were not analyzed. Third, non-surgical or palliative surgery patients were not collected, and the relationship between tumor characteristics and prognosis of such patients and serum inflammatory indexes could not be assessed. Fourth, the role and prognosis of immune cell infiltration may vary depending on the site of infiltration, such as infiltrating margins or interstitium.

## Conclusions

Preoperative LMR, a pure inflammatory marker, was an independent prognostic factor for OS in HCCA patients. We observed that low LMR correlated with low levels of tumor-infiltrating CD3^+^ T cells. In addition, HCCA patients with low LMR and CD3^+^ T cells infiltration had a worse prognosis for postoperative survival compared to others. Additionally, this study suggested the possibility that the presence of TILs in the tumor microenvironment could be evaluated noninvasively with markers in peripheral blood samples. Overall, this study suggests that patients with operable HCCA with lower preoperative peripheral blood LMR levels have a worse prognosis and require more aggressive follow-up and treatment. In addition, HCCA patients with lower peripheral blood NLR have more infiltrating CD8+ T cells in their tumors and may be more appropriate for treatment with immune checkpoint inhibitors.

## Data Availability Statement

The original contributions presented in the study are included in the article/**Supplementary Material**. Further inquiries can be directed to the corresponding authors.

## Ethics Statement

The studies involving human participants were reviewed and approved by Ethics Committee of the First Affiliated Hospital of Dalian Medical University. Written informed consent for participation was not required for this study in accordance with the national legislation and the institutional requirements.

## Author Contributions

GT and ZN conceived the project, and GT supervised the project. Z-qL and CM designed and performed most of analysis. W-zC provided significant intellectual input. Z-qL and ZN wrote the manuscript with input from all other authors. All authors contributed to the article and approved the submitted version.

## Funding

This study is supported by the National Natural Science Foundation of China grants (No. 81972217 and No. 81502024), Key Research and Developmentprogram of Liaoning Province (2019JH8/10300029) and Project funded by Dalian Science and Technology Bureau (2019RQ074).

## Conflict of Interest

The authors declare that the research was conducted in the absence of any commercial or financial relationships that could be construed as a potential conflict of interest.

## Publisher’s Note

All claims expressed in this article are solely those of the authors and do not necessarily represent those of their affiliated organizations, or those of the publisher, the editors and the reviewers. Any product that may be evaluated in this article, or claim that may be made by its manufacturer, is not guaranteed or endorsed by the publisher.
